# Long-range optical trapping and binding of microparticles in hollow-core photonic crystal fibre

**DOI:** 10.1038/s41377-018-0015-z

**Published:** 2018-06-20

**Authors:** Dmitry S. Bykov, Shangran Xie, Richard Zeltner, Andrey Machnev, Gordon K. L. Wong, Tijmen G. Euser, Philip St.J. Russell

**Affiliations:** 1 Max Planck Institute for the Science of Light, Staudtstr. 2, 91058 Erlangen, Germany; 2 Department of Physics, University of Erlangen-Nuremberg, 91058 Erlangen, Germany; 3 NanoPhotonics Centre, Cavendish Laboratory, University of Cambridge, Cambridge, CB3 0HE UK

## Abstract

Optically levitated micro- and nanoparticles offer an ideal playground for investigating photon–phonon interactions over macroscopic distances. Here we report the observation of long-range optical binding of multiple levitated microparticles, mediated by intermodal scattering and interference inside the evacuated core of a hollow-core photonic crystal fibre (HC-PCF). Three polystyrene particles with a diameter of 1 µm are stably bound together with an inter-particle distance of ~40 μm, or 50 times longer than the wavelength of the trapping laser. The levitated bound-particle array can be translated to-and-fro over centimetre distances along the fibre. When evacuated to a gas pressure of 6 mbar, the collective mechanical modes of the bound-particle array are able to be observed. The measured inter-particle distance at equilibrium and mechanical eigenfrequencies are supported by a novel analytical formalism modelling the dynamics of the binding process. The HC-PCF system offers a unique platform for investigating the rich optomechanical dynamics of arrays of levitated particles in a well-isolated and protected environment.

## Optical trapping: multiple particle binding

Microparticles placed inside the evacuated hollow core of a photonic crystal fibre can be bound together by optical forces and moved as a single entity along the fibre by light. Dmitry Bykov and coworkers from the Max Planck Institute for the Science of Light in Erlangen, Germany performed the experiments with three, 1 µm-diameter polystyrene particles. When light pulses from a Ti:Sa laser were injected into both ends of the photonic crystal fibre, intermodal interference was found to be able to bind the three particles together, with a particle spacing of ~40 µm. It is thought that by adjusting parameters such as the fibre design and the laser pulse duration, chirp and power it should be possible to increase both the inter-particle binding distance and the number of bound particles.

## Introduction

Since Ashkin’s first report of the acceleration and trapping of microparticles by optical forces^1^, the use of optical tweezers has developed into a standard technique for biological manipulation and pico-Newton force sensing^2,3^, to mention just two examples from a wide range of applications. In recent years, the emerging field of “levitated optomechanics” has attracted increasing interest. An optically tweezered particle, especially at low gas pressure, is isolated from the external environment, resulting in very low mechanical damping. This leads to very-high mechanical Q-factors and permits particle rotational speeds in the MHz range^4,5^. Recent advances include the use of feedback or cavity cooling of the centre-of-mass temperature towards the mechanical ground state^6–11^, permitting experimental tests of fundamental physics^12–14^, and the use of individual levitated particles as point sensors^15,16^. Optical binding between arrays of trapped particles adds an additional dimension, and will result in rich dynamics, enabling access to collective coupling between high-Q mechanical oscillators and, potentially, simultaneous cooling of the mechanical motion of multiple particles.

Multiple trapping sites have previously been created using interference^17^ and holographic tweezers^18^, allowing the formation of a lattice of trapped microparticles. In these experiments, there is typically very-little multiple scattering between particles—a necessary prerequisite for optical binding^19–22^, which can only occur if the scattered field from one particle strongly interacts with the other particles in the array, and vice-versa. In a 1D particle array, such as the one studied here, binding is possible because the optical fields propagate bidirectionally along the array.

The majority of 1D optical binding experiments to date have been performed in free space over distances that are limited by the Rayleigh range of the focusing optics. However, to control and measure the collective binding dynamics of such arrays, it is necessary to manipulate and monitor individual particles within the array without perturbing the other degrees of freedom, which requires extended inter-particle distances and, therefore, long-range interactions between trapped particles. In recent years, the optical binding range has been extended using non-diffracting Bessel beams^21^ or by trapping particles in the evanescent field at the surface of a multimode glass microfibre^23^. These experiments were however conducted in a liquid environment, resulting in strongly damped collective dynamics. In evanescent field-based traps, particles are in physical contact with the microfibre (not levitating), giving rise to additional damping terms^23^. In addition, external spatial light modulators were used in those experiments to create binding sites, limiting the stability and power handling of the system.

Here we report long-range optical binding of a chain of levitated particles inside the core of an evacuated hollow-core photonic crystal fibre (HC-PCF). When the fundamental mode is launched into the HC-PCF, particle-induced scattering to higher-order guided modes results in an intermodal interference pattern, and a landscape of periodically distributed trapping potentials, within which subsequent particles can be trapped. The binding forces vary as the particles move relative to each other, and stable trapping and binding of a chain of particles results for configurations that locally minimise the free energy of the system. By offering modal fields that are tightly confined over the entire fibre length, i.e., an infinite Rayleigh length, HC-PCF permits trapped particles to interact over distances orders of magnitude longer than the trapping wavelength, limited only by fibre and scattering loss. The absence of viscous damping at low gas pressure makes it possible to observe, for the first time, the collective dynamics of a bound-particle chain—an important first step towards “levitated collective optomechanics”.

## Materials and methods

**Fig. 1:**
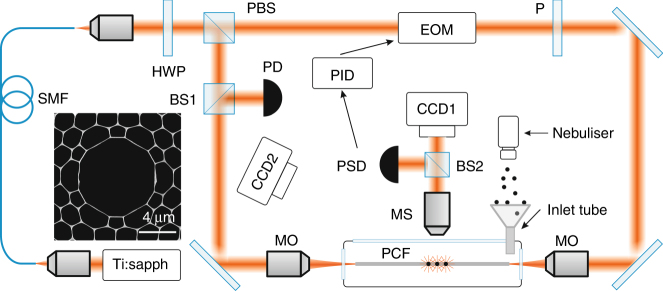
Schematic of the experimental set-up. SMF, single mode fibre; HWP, half-wave plate; PBS, polarising beam splitter; MO, microscope objective; PD, photodiode; MS, microscope system; PSD, position-sensing diode; PID, proportional-integral-differential controller; EOM, electro-optical modulator; P, polarizer. Inset: scanning electron micrograph of the HC-PCF

The experimental set-up is sketched in Fig. 1. Light from a pulsed Ti:sapphire laser (800 nm wavelength, 80 MHz repetition rate, 60 fs 1/e half-width pulse duration) was delivered to the set-up through a 15 m long single mode fibre (SMF, with group velocity dispersion *β*_2_ = 40 fs/(m.THz)). This caused the pulses to broaden to 1/e half-width durations of ~14.4 ps at the output of the SMF, with a frequency chirp of ~0.5 THz/ps. The pulses were split at a polarising beam splitter (PBS) and coupled into the opposite ends of an 8 cm length of HC-PCF with a core diameter of 7.8 µm, which was mounted inside a vacuum chamber. The counter propagating trapping beams were orthogonally polarised to prevent the formation of intensity interference fringes. A 96:4 beam splitter (BS1) and photodiode (PD) were used to monitor the transmitted power at one end of the HC-PCF. A medical nebuliser was used to inject polystyrene particles (typical diameters of 1 µm) into a dual-beam trap placed close to the fibre input face^24^ (see Supplementary Information S1). Once trapped, a particle was propelled into the hollow core by adjusting the beam splitter to increase the forward power. Subsequent particles were launched into the fibre using the same technique (see Supplementary Media S1). Once the desired number of particles had been launched, the chamber was sealed and pumped out to a pressure of a few mbar.

## Results and discussion

When light is launched into the HC-PCF, it is difficult to avoid weak excitation of higher-order modes (HOMs). Even a few percent of HOM power will cause strong intermodal interference, which, however, fades away with distance due to a combination of the group velocity walk-off and pulse chirp. Thus, when a single particle is launched into the core, the transmitted power will fluctuate as the particle passes through the interference pattern, eventually stabilising once the particle reaches a position where the stationary interference between the HOM pulses has faded away. The fade-away length can be written in the form (see Supplementary Information S2):

1 $$z_{\mathrm{F}} = \frac{{\bar v_{\mathrm{G}}\tau _0}}{2}\sqrt {\frac{{\bar v_{\mathrm{G}}\pi \left( {1 + (L/L_{\mathrm{D}})^2} \right)}}{{\Delta v_{\mathrm{G}}(L/L_{\mathrm{D}})}}}$$

where *L*_D_ = τ_0_^2^/|*β*_2_| is the dispersion length in the SMF, *τ*_0_ is the 1/e half-width pulse duration at the laser, *L* is the SMF length, Δv_G_ is the group velocity difference between the HC-PCF modes and $\bar v_{\mathrm{G}}$ is the mean group velocity (spectral broadening due to self-phase modulation in the SMF has been neglected, but is expected to be small). For the experimental parameters, *z*_F_ = 1.5 mm.

**Fig. 2:**
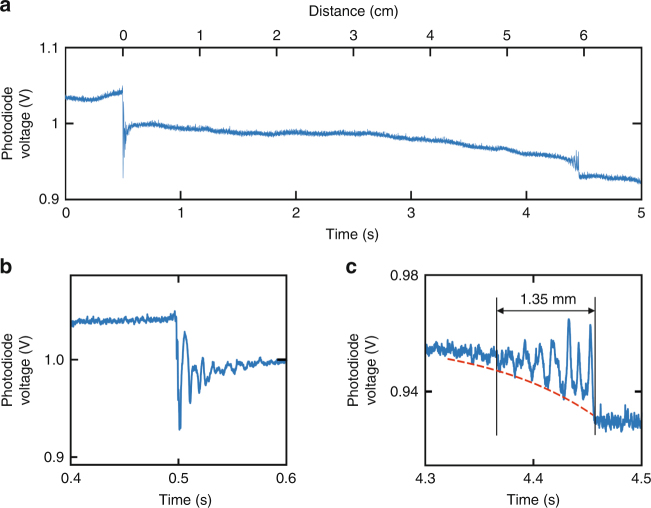
Intermodal interference induced by trapped particles inside the HC-PCF. **a** With one particle already loaded inside the HC-PCF, the temporal variation in transmitted power is measured as the second particle enters the fibre and approaches the first one. **b** Zoom into the time interval between 0.4 and 0.6 s, showing the fade-out of intermodal beating around the fibre endface. **c** Zoom into the time interval from 4.3 to 4.5 s, when the second particle approaches the first one. The red-dashed line is an exponential fit to the intermodal beating envelope

A very similar effect is seen when two or more particles are close enough to sit in their respective intermodal interference patterns. Under these circumstances long-range optical binding can be observed. To explore these effects, we launched a 1-μm-diameter polystyrene particle into the fibre, followed by a second similar particle 0.5 s later (Fig. 2a, b). The power ratio was adjusted so that the second particle moved towards the first one. The transmitted power at the photodiode is plotted against time over a 5 s interval in Fig. 2a. The effects of intermodal interference are seen at 0.5 s and 4.4 s, with magnifications of the responses in each case shown in Fig. 2b, c. As expected, intermodal interference causes the transmitted power to oscillate as the second particle moves away from the input face (Fig. 2b). After 0.55 s, the transmitted power becomes constant. Then, at 4.36 s, the first particle begins to be disturbed by the intermodal interference created by the second particle (Fig. 2c). The oscillations increase in amplitude until the two particles become stably bound, when the modulation in the transmitted signal once again becomes constant. An exponential fit (dashed-red curve) to the envelope of the oscillations yields a decay time of ~0.9 s, which, given that the average speed of the particles is ~1.5 cm/s, corresponds to a fade-out distance of ~1.35 mm, in good agreement with the above analysis.

**Fig. 3:**
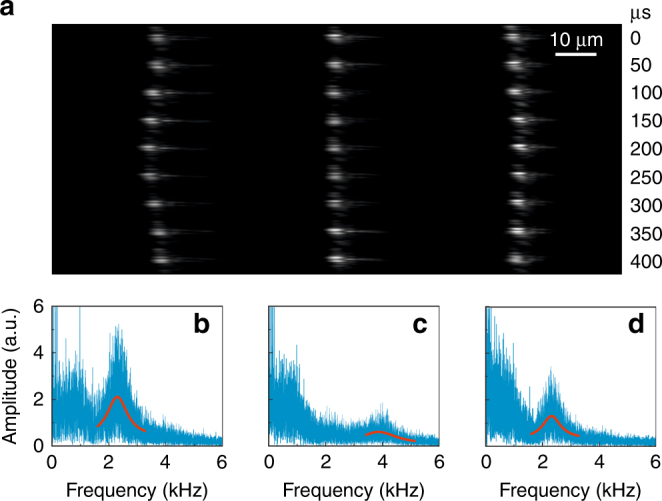
Thermally driven collective motion of the bound-particle array. **a** A series of snapshots of the bound-particle array during one period of the breathing mode, captured with a high-speed camera. **b–d** Spectra for the mechanical motion of the three bound particles. The blue curves are the measured data, and the red curves are the Lorentzian fits

Three particles were then launched into the fibre in sequence, and after several attempts and adjustments, it was found that these particles were optically bound. Figure 3a shows optical images of the bound-particle array at equilibrium, captured through the side of the HC-PCF by using a CCD camera. The particles are spaced by 40 ± 3 μm, and the uncertainty is caused by the finite width of the intensity peaks at the CCD. Assuming that there are several elementary charges on each particle^11^, we estimate the inter-particle Coulomb forces to be several atto-Newton, which will displace the particles from their equilibrium positions by only a few tens of femtometres, which is negligible compared to the inter-particle distance.

Thermally driven vibrations for the bound-particle array (driven by Brownian motion) were resolved at a pressure of 6 mbar by using a high-speed video camera (20,000 frames per sec). Figure 3a shows 9 consecutive frames at time intervals of 50 µs (see Supplementary Media S2 for the video). During the measurement, electronic feedback was used to stabilise the particle array within the field of view of the imaging system using a position-sensing diode (PSD) to generate a signal proportional to the centre-of-mass motion of the particles. This was then fed back via a PID controller to the EOM.

The “breathing” mode of the bound particles, in which the two outer particles move out-of-phase while the central particle is stationary, can be directly observed in the video frames (Fig. 3a). The peak-to-peak amplitude for the vibrations is ~5 µm according to the scale bar in the video. The spectra for the thermally driven centre-of-mass motion of particles 1, 2 and 3 are plotted in Fig. 3b-d using data extracted from the high-speed video. The red-solid curves are Lorentzian fits to the individual spectral peaks. For particles 1 and 3, a strong peak occurs at 2.3 kHz (the frequency of the breathing mode), while for particle 2, a much weaker peak appears at 3.9 kHz, related to the case in which the central particle moves out-of-phase with the two outer particles (see the analysis below). Note that these distinct resonances can only be resolved at a low gas pressure, when gas-related viscous damping is suppressed.

Experimentally up to five particles have been successfully bound to form an array moving to-and-fro along the fibre (see Supplementary Media S3 for the video). Here, using three bound particles as an example, we develop scattering matrix analysis to calculate the forces acting on each particle, and, thus, identify configurations that result in stable binding. We represent the complex amplitudes of individual modes in the system by a column vector **v**, which is normalised so that v·v* = 1, meaning that the power in the *i*-th mode is _*Pi*_ = |**v**_*i*_|^2^*P*_0_, where *P*_0_ is the total power. The modes are assumed to form a complete orthogonal set. Particle-induced scattering between incident and transmitted modes can then be described by **v**_out_ = [**S**]·**v**_in_, where [**S**] is the scattering matrix and **v**_in_ and **v**_out_ are column vectors, the elements of which are the complex amplitudes of the incident and scattered modes. Orthogonality allows us to write the scattering coefficient from incident mode *i* to forward-scattered mode *j* in the form:

2 $$S_{ji} = \frac{{\mathop {\int \hskip-4pt \int}\nolimits_A {s_im_j^ \ast dA} }}{{\mathop {\int \hskip-4pt \int}\nolimits_A {\left| {m_i} \right|^2dA} }}$$

where integration is over the transverse plane, *m*_*i*_(*x*, *y*) is the transverse field distribution of the *i*-th mode and *s*_*i*_(*x*, *y*) is the scattered field distribution immediately after the particle, which we calculate by 3D finite element modelling.

**Fig. 4:**
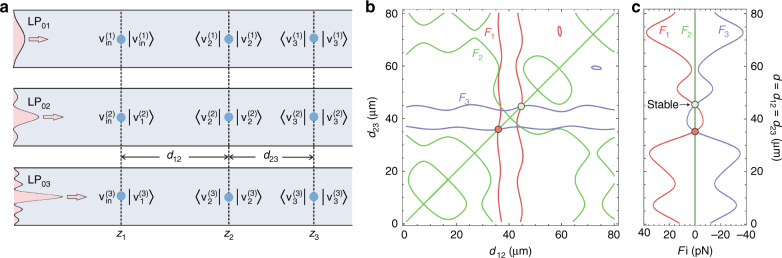
Scattering matrix analysis of the binding process. **a** Sketch of the scattering matrix analysis illustrating the notation used. **b** Plots of the loci along which the forces on the particles are zero. Red: particle 1, green: particle 2 and blue: particle 3. Stable and unstable trapping points when the forces on all three particles are zero are indicated by green and red dots, respectively. **c** Optical forces acting on the three particles as a function of the inter-particle distance *d* when the central particle is stationary

We now assume that back-scattering (which is very weak for the experimental parameters) and material absorption are negligible. We also assume that the particle is trapped at the centre of the core (this seems to be a good approximation since the launching beam is Gaussian) so that scattering occurs only to radially symmetric modes and that the Coulomb forces between the particles and core wall are negligible. Figure 4a shows the geometry of the three-particle system. The phase index, group index and loss of the modes were calculated by finite element modelling of the actual fibre microstructure based on a high-resolution scanning electron micrograph. Since linearly polarised LP_0*i*_ modes of the order *i* > 3 were found to have a very-high propagation loss, contributing negligibly to optical binding, we included only the first three lowest-order modes (*i* = 1, 2, 3), resulting in a 3 × 3 scattering matrix.

Since binding is observed at ~4 cm from the fibre input and the fade-out distance of intermodal beating is ~1.35 mm, the three incident modes will each independently transfer momentum to the particles because their instantaneous frequencies at each position along the fibre are different and no stationary intermodal beat-pattern can form. Additionally, since the pulse durations (ps) are orders of magnitude shorter than the mechanical response time of the particle (hundreds of μs), the particles will respond to the vector sum of the resulting optical momenta. Thus, each incident mode can be treated independently. For the *p*-th incident mode, the modal amplitudes on opposite sides of the first particle are (using bracket notation to indicate left and right, respectively):

3 $$\left\langle {{\mathbf{v}}_1^{\left( p \right)}} \right| = {\mathbf{v}}_{{\mathrm{in}}}^{\left( p \right)} , \left| {{\mathbf{v}}_1^{\left( p \right)}} \right\rangle = [{\mathbf{S}}]\left\langle {{\mathbf{v}}_1^{\left( p \right)}} \right|$$

for the second particle:

4 $$\left\langle {{\mathbf{v}}_2^{\left( p \right)}} \right| = [{\mathbf{P}}_{21}][{\mathbf{S}}]{\mathbf{v}}_{{\mathrm{in}}}^{\left( p \right)},\,\left| {{\mathbf{v}}_2^{\left( p \right)}} \right\rangle = [{\mathbf{S}}]\left\langle {{\mathbf{v}}_2^{\left( p \right)}} \right|$$

and for the third particle:

5 $$\left\langle {{\mathbf{v}}_3^{\left( p \right)}} \right| = [{\mathbf{P}}_{32}][{\mathbf{S}}][{\mathbf{P}}_{21}][{\mathbf{S}}]{\mathbf{v}}_{{\mathrm{in}}}^{\left( p \right)} ,\,\left| {{\mathbf{v}}_3^{\left( p \right)}} \right\rangle = [{\mathbf{S}}]\left\langle {{\mathbf{v}}_3^{\left( p \right)}} \right|$$

The propagation matrix [**P**_*kl*_] between particle *l* and particle *k* is diagonal, with elements (1, exp[*iβ*_12_(*z*_*k*_−*z*_*l*_)], exp[*iβ*_12_(*z*_*k*_−*z*_*l*_)]), where *β*_*pq*_ = *β*_*p*_–*β*_*q*_ is the propagation constant difference between the LP_0*p*_ and LP_0*q*_ modes (*p* < *q*). The same analysis applies for the backward-propagating modes. The launched amplitudes of the three modes at the fibre input were estimated by calculating their overlap with a focused Gaussian beam (beam waist ~2.5 μm), yielding ${\mathbf{v}}_{{\mathrm{in}}}^{(1)} = (0.982,0,0)$, ${\mathbf{v}}_{{\mathrm{in}}}^{(2)} = (0,0.143,0)$ and ${\mathbf{v}}_{{\mathrm{in}}}^{(3)} = (0,0,0.081)$.

To calculate the axial optical force $\vec F_k^p$ on the *k*-th particle, for the incidence of the *p*-th mode on the first particle, we first need to find the local intensity on each side of the particle. This involves taking the modulus squared of the sum of the local field amplitudes. The total optical force on particle *k* exerted by forward-travelling light is then given by:

6 $$\vec F_k = \frac{{P_0}}{{2c}}\mathop {\sum}\limits_{p = 1}^3 {\left( {\left| {{\mathbf{N}} \cdot \left\langle {{\mathbf{v}}_k^{\left( p \right)}} \right|} \right|^2 - \left| {{\mathbf{N}} \cdot \left| {{\mathbf{v}}_k^{\left( p \right)}} \right\rangle } \right|^2} \right)}$$

where *P*_0_/2 is the total power incident in the forward direction (an equal amount is incident in the backward direction) and *c* is the speed of light *in vacuo*. The three-element row-matrix **N** has elements that correct for the overlap between the particle and individual mode shapes:

7 $$N_i = \sqrt {{\int}_0^{a_{\mathrm{p}}} {{\mathrm{J}}_0^2(u_{0i}r{\mathrm{/}}a)} 2\pi rdr{\mathrm{/}}{\int}_0^a {{\mathrm{J}}_0^2(u_{0i}r{\mathrm{/}}a)} 2\pi rdr}$$

where *a* is the core radius, *a*_p_ is the particle radius and *u*_0*i*_ is the *i*-th zero of the Bessel function J_0_. For our experimental parameters, *N*_1_ = 0.250, *N*_2_ = 0.362 and *N*_3_ = 0.412. The total optical force on particle *k* is found by adding the complementary force $\overleftarrow F_k$ exerted by the backward-propagating light (see Supplementary Information S3).

A necessary (but insufficient) condition for binding occurs when the optical forces acting on all three particles are simultaneously zero. Figure 4b plots the lines of zero optical force on each particle as a function of the inter-particle distances *d*_12_ and *d*_23_. The diagonal line represents the symmetric configuration when *d*_12_ = *d*_23_ = *d*, and Fig. 4c plots the three forces vs. *d* in this case. All three forces are simultaneously zero at two positions, one of which is unstable (red dot) and the other is stable (green dot). This predicts an inter-particle binding distance of *d* = 44.6 μm, in good agreement with the measurements. These binding positions are found to be relatively insensitive to the launched beam waist at each end of the fibre (in agreement with experiment), i.e., a change in beam waist from 2.5 to 2 μm results in the predicted inter-particle distance varying from 44.6 to 48.1 μm.

The stiffness of the optical springs in each particle trap can be straightforwardly calculated by partial differentiation of the forces with respect to the particle displacement, resulting in a stiffness tensor [**K**]:

8 $$[{\mathbf{K}}] = \left( {\begin{array}{*{20}{c}} {k_{11}} & {k_{12}} & {k_{13}} \\ {k_{21}} & {k_{22}} & {k_{23}} \\ {k_{31}} & {k_{32}} & {k_{33}} \end{array}} \right) = \left( {\begin{array}{*{20}{c}} { - \frac{{\partial F_1}}{{\partial z_1}}} & { - \frac{{\partial F_1}}{{\partial z_2}}} & { - \frac{{\partial F_1}}{{\partial z_3}}} \\ { - \frac{{\partial F_2}}{{\partial z_1}}} & { - \frac{{\partial F_2}}{{\partial z_2}}} & { - \frac{{\partial F_2}}{{\partial z_3}}} \\ { - \frac{{\partial F_3}}{{\partial z_1}}} & { - \frac{{\partial F_3}}{{\partial z_2}}} & { - \frac{{\partial F_3}}{{\partial z_3}}} \end{array}} \right) \\ = \left( {\begin{array}{*{20}{c}} { - 0.10} & {0.08} & {0.016} \\ {0.13} & { - 0.26} & {0.13} \\ {0.016} & {0.08} & { - 0.10} \end{array}} \right)pN/\mu m$$

The components of the tensor are numerically calculated using the above model for *d*_12_ = *d*_23_ = *d* and *P*_0_ = 50 mW. The asymmetry of [**K**], e.g., $k_{12} \ne k_{21}$, is caused by the fact that particles 1 and 2 scatter differently to particle 3, so that for the same values of displacement ($\Delta z_1 = \Delta z_2$), $\Delta F_2 \ne \Delta F_1$. From Eq. (8) we can obtain the equation of motion for the collective oscillations $\ddot A(t) + [{\mathbf{M}}]^{ - 1}[{\mathbf{K}}] \cdot A(t) = {\mathbf{0}}$, where [**M**] is a diagonal matrix with elements given by the mass of each particle (5.86 × 10^–16^ kg). The mechanical frequencies and shapes of the eigenmodes can then be calculated (Table 1), where *A*_k_ represents the oscillation amplitude of the *k*-th particle of the corresponding eigenmode. The calculated eigenfrequencies agree with the observations with no free parameters. This theoretical model may be extended to analyse the case with more than three bound particles and to study the collective dynamics with external driving or dissipative terms.

**Table 1:** Frequencies and shapes for the mechanical modes

Ω/2π (kHz)	*A*_1_	*A*_2_	*A*_3_
3.85	–0.30	0.91	–0.30
2.24	–0.71	0	0.71
0	0	0	0

## Conclusions

In summary, multiple polystyrene microparticles can be optically bound by intermodal interference within the evacuated core of a HC-PCF, where protected from environmental disturbance, their collective vibrational modes can be resolved. By adjusting the trapping pulse chirp and duration as well as the fibre dispersion, the binding length (40 µm in the experiments reported here) can be increased, potentially allowing particle binding over centimetre distances. By further increasing the average power of the pulsed trapping laser, it will be possible to achieve stable trapping and binding of particles tens of nanometre in diameter and to increase the number of bound particles within the array. Currently the lowest gas pressure is set by the increasing instability of optically trapped particles in vacuum^6,12–14^. By using electronic feedback to stabilise the mechanical motion at ultralow pressure, it should be possible to reach higher mechanical Q-factors and to explore cooling to the ground-state, nonlinear coupling and synchronisation of the motion of multiple particles. Finally, the whole assembly of particles can be moved to-and-fro along the HC-PCF, which may enable potential applications in remote sensing, with the bound-particle array being propelled into a region where external perturbations are present. A bound-particle array offers more mechanical degrees of freedom than a single trapped particle in a HC-PCF, suggesting more complex forms of flying particle sensors^15^. In addition, versatile multi-sensors can be constructed by binding particles with different physical properties.

## Electronic supplementary material

Supplementary information(DOCX 109 kb)

MediaS1_ParticleLaunching

MediaS2_BreathingMode

MediaS3_BoundArrayWithFiveParticles
