# Supplementary material for: Long-range optical trapping and binding of microparticles in hollow-core photonic crystal fibre
Source: Light Sci Appl. 2018 Jun 20;7:22. doi: 10.1038/s41377-018-0015-z (PMC6107024; doi:10.1038/s41377-018-0015-z)
Supplement: Supplementary file 1 — Supplementary information(DOCX 109 kb) [file 41377_2018_15_MOESM1_ESM.docx]

Long-range optical trapping and binding of microparticles in hollow-core photonic crystal fibre

Supplementary Information

Dmitry S. Bykov^1^, Shangran Xie^1,*^, Richard Zeltner^1,2^, Andrey Machnev^1^, Gordon K. L. Wong^1^,

Tijmen G. Euser^1,3^, and Philip St.J. Russell^1,2^

^1^Max Planck Institute for the Science of Light, Staudtstr. 2, 91058 Erlangen, Germany
^2^Department of Physics, University of Erlangen-Nuremberg, 91058 Erlangen, Germany
^3^NanoPhotonics Centre, University of Cambridge, Cavendish Laboratory, CB3 0HE Cambridge, UK

*shangran.xie@mpl.mpg.de

**S1. Procedure of trapping particles in front of the HC-PCF**

The aerosol launching procedure described in Ref. 1 was used to trap the particle in front of the HC-PCF: the particles were first dissolved in water in a particle-to-water mass ratio of 10^-3^–10^-2^. A medical nebulizer was used to produce droplets of the particle solution, with each droplet containing a single particle on average. The droplets were injected into the chamber via an inlet tube in the lid that was placed above-in-front of the fibre until one of them was trapped in front of the HC-PCF core. The laser irradiation usually evaporates the droplet within a few seconds, leaving the particle in the dual-beam optical trap.

A trapping event can be observed either via dropping of the transmitted power (from fractions of percent for 100 nm particle up to 5% for 1 µm particle) or by observing changes in the image of the fibre endface taken by an external camera (CCD2 in Fig. 1a). As shown in Media S1, the image of the fibre endface starts to wobble a bit due to Brownian motion of the trapped particle. Bigger particles can be launched by using a glass plate mounted on a piezoelectric transducer which is placed below the fibre endface. The piezoelectric transducer can be driven at the resonance frequency of the glass plate, catapulting the particles in front of the fibre.

**S2. Analysis of pulse walk-off distance inside HC-PCF**

In our experiment, the pulsed Ti:sapphire laser (80 MHz repetition rate, 60 fs 1/e half-width pulse duration) was delivered to the set-up through a 15 m length of SMF. The in-coupling to the HC-PCF and the trapped particle would excite HOMs with different group velocities. The walk-off distance of the pulsed trapping beam inside HC-PCF therefore contributed from both the pulse chirping in SMF and the modal walk-off in HC-PCF. Assuming a Gaussian pulse from the initial Ti:sapphire laser with field amplitude written as

$E\left(\tau\right)=\exp\left(-\tau^{2}/\left(2\tau_{0}^{2}\right)\right)$ (S1)

where *τ* is the time frame, *τ*_0_ is the 1/e half-width. The pulse envelop after propagating a distance *L* along the SMF with group velocity dispersion *β*_2_ is^2^

$E\left(\tau,L\right)=\frac{\tau_{0}}{\sqrt{\tau_{0}^{2}-i\beta_{2}L}}\exp\left(\frac{-\tau^{2}}{2\left(\tau_{0}^{2}-i\beta_{2}L\right)}\right)$ (S2)

with 1/e half-width given by

$\tau_{1/2}=\tau_{0}\sqrt{1+\beta_{2}^{2}L^{2}/\tau_{0}^{4}}=\tau_{0}\sqrt{1+L^{2}/L_{D}^{2}}$ (S3)

where *L*_D_ is the dispersion length. Given *β*_2_ = 40 fs/(m.THz) for SMF and *L* = 15 m, the 1/e half-width of the chirped pulse at the output end of the SMF is ~14.4 ps. The instantaneous frequency deviation from the carrier frequency *ω*_0_ is given by

$\delta\omega\left(\tau,L\right)=\frac{2L/L_{D}}{1+\left(L/L_{D}\right)^{2}}\frac{\tau}{\tau_{0}^{2}}$ (S4)

Inside HC-PCF, the excited LP_0_*_i_* modes will walk off from each other, with the walk-off time$\Delta\tau=z\Delta\nu_{G}/\bar{\nu}_{G}^{2}$, where *z* is propagation distance inside HC-PCF, Δ*v*_G_ the group velocity difference between the modes and *v*_G_ is the mean modal group velocity. Therefore the several modal pulses would have different frequencies, with the frequency offset taking the value:

$\Delta f=\frac{2L/L_{D}}{1+\left(L/L_{D}\right)^{2}}\frac{z\Delta\nu_{G}}{\pi\bar{\nu}_{G}^{2}\tau_{0}^{2}}$ (S5)

Here we ignore the weak group velocity dispersion of HC-PCF compared to SMF. Intermodal beating will fade away mainly due to temporal oscillations that average the beating to zero. This will occur approximately when $\Delta f\cdot z/\bar{\nu}_{G}\leq1/2$, which means that intermodal beating will fade out after a distance:

$z_{\text{F}}=\frac{\bar{v}_{\text{G}}\tau_{0}}{2}\sqrt{\frac{\bar{v}_{\text{G}}\pi\left(1+(L/L_{\text{D}})^{2}\right)}{\Delta v_{\text{G}}(L/L_{\text{D}})}}$ (S6)

which is identical to Eq. (1) in the primary manuscript.

**S3. Optical force induced by the backward-propagating light**


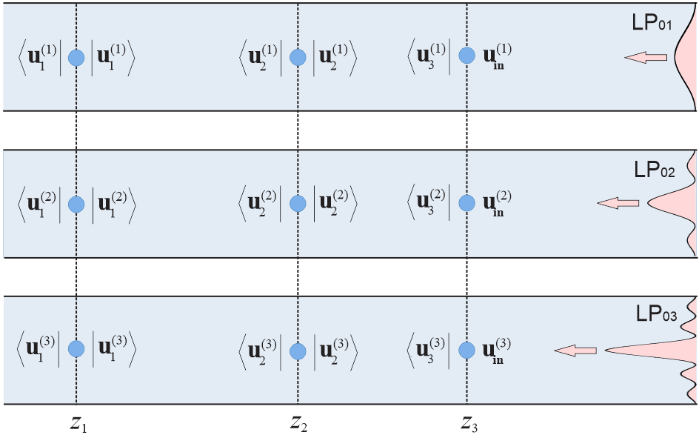


Fig. S1. Sketch of the scattering matrix analysis for the backward-propagating light.

Following the analysis of forward-propagating light in the primary manuscript, Fig. S1 sketches the configuration and notation for the backward-propagating light. Here we use the column vector **u** to represent the complex amplitudes of fibre modes. In this case, for the *p*-th incident mode, the modal amplitudes on opposite sides of the particles are:

$|\mathbf{u}_{3}^{\left(p\right)}\rangle=\mathbf{u}_{\text{in}}^{\left(p\right)} , \langle\mathbf{u}_{3}^{\left(p\right)}|=[\mathbf{S}]|\mathbf{u}_{3}^{\left(p\right)}\rangle$ (S7)

for the third particle:

$|\mathbf{u}_{2}^{\left(p\right)}\rangle=[\mathbf{P}_{23}][\mathbf{S}]\mathbf{u}_{\text{in}}^{\left(p\right)}, \text{ }\langle\mathbf{u}_{2}^{\left(p\right)}|=[\mathbf{S}]|\mathbf{u}_{2}^{\left(p\right)}\rangle$ (S8)

for the second and :

$|\mathbf{u}_{3}^{\left(p\right)}\rangle=[\mathbf{P}_{12}][\mathbf{S}][\mathbf{P}_{23}][\mathbf{S}]\mathbf{u}_{\text{in}}^{\left(p\right)} ,\text{ }\langle\mathbf{u}_{3}^{\left(p\right)}|=[\mathbf{S}]|\mathbf{u}_{3}^{\left(p\right)}\rangle$ (S9)

for the first. [**P***_kl_*] is defined in the primary manuscript, and$\mathbf{u}_{\text{in}}^{(p)}=\mathbf{v}_{\text{in}}^{(p)}$. The optical force contributed from the backward-propagating light shares the same form of Eq. (6) of the primary manuscript except replacing **v** with **u**:

$\overleftarrow{F}_{k}=\frac{P_{0}}{2c}\sum_{p=1}^{3} \left(\left|\mathbf{N}\cdot\langle\mathbf{u}_{k}^{\left(p\right)}|\right|^{2}-\left|\mathbf{N}\cdot|\mathbf{u}_{k}^{\left(p\right)}\rangle\right|^{2}\right)$ (S10)

The total optical force acting on particle *k* is the sum of the forward and backward contributions, thus:

$F_{k}=\vec{F}_{k}+\overleftarrow{F}_{k}$ (S11)

**Media S1:** Launching multiple particles inside HC-PCF

**Media S2:** Breathing modes of the bound-particle array

**Media S3:** Moving bound-array with five particles

References

1. Summers MD, Burnham DR, McGloin D. Trapping solid aerosols with optical tweezers: A comparison between gas and liquid phase optical traps. Opt Express 2008; 16: 7739.
2. Agrawal GP. Nonlinear fibre optics 5th ed. Ch. 3.2.1 (Elsevier, 2013).
